# The Paget's disease of bone risk gene *PML* is a negative regulator of osteoclast differentiation and bone resorption

**DOI:** 10.1242/dmm.049318

**Published:** 2022-04-26

**Authors:** Sachin Wani, Anna Daroszewska, Donald M. Salter, Rob J. van ‘t Hof, Stuart H. Ralston, Omar M. E. Albagha

**Affiliations:** 1 Rheumatology and Bone Disease Unit, Centre for Genomic and Experimental Medicine, MRC Institute of Genetics and Cancer, University of Edinburgh, Edinburgh EH4 2XU, UK; 2 Institute of Life Course and Medical Sciences, University of Liverpool, Liverpool L7 8TX, UK; 3 Vanthof Scientific, Torun 87-100, Poland; 4 College of Health and Life Sciences, Hamad Bin Khalifa University, Doha, P.O. Box 34110, Qatar

**Keywords:** Bone, PML, Paget's disease, Osteoclasts

## Abstract

Paget's disease of bone (PDB) is characterized by focal increases in bone remodelling. Genome-wide association studies identified a susceptibility locus for PDB tagged by rs5742915, which is located within the *PML* gene. Here, we have assessed the candidacy of *PML* as the predisposing gene for PDB at this locus*.* We found that the PDB-risk allele of rs5742915 was associated with lower *PML* expression and that *PML* expression in blood cells from individuals with PDB was lower than in controls. The differentiation, survival and resorptive activity of osteoclasts prepared from *Pml^−/−^* mice was increased compared with wild type. Furthermore, the inhibitory effect of IFN-γ on osteoclast formation from *Pml^−/−^* was significantly blunted compared with wild type. Bone nodule formation was also increased in osteoblasts from *Pml^−/−^* mice when compared with wild type. Although microCT analysis of trabecular bone showed no differences between *Pml^−/−^* mice and wild type, bone histomorphometry showed that *Pml^−/−^* mice had high bone turnover with increased indices of bone resorption and increased mineral apposition rate. These data indicate that reduced expression of PML predisposes an individual to PDB and identify PML as a novel regulator of bone metabolism.

This article has an associated First Person interview with the first author of the paper.

## Abstract (precis)

**Summary:** *PML*, a susceptibility gene for Paget's disease of bone, is a novel negative regulator of bone metabolism. The differentiation of osteoclasts and osteoblasts is enhanced in *Pml^−/−^* mice compared with controls.

## INTRODUCTION

Paget's disease of bone (PDB) is a skeletal disorder characterised by focal increases in disorganised bone remodelling with markedly increased osteoclast and osteoblast activity. Commonly affected sites include the pelvis, femur, lumbar spine, skull and tibia (Gennari et al., 2019; Ralston et al., 2019). Many patients are asymptomatic, but others suffer from various complications, including bone pain, bone deformity, deafness and secondary osteoarthritis (Tan and Ralston, 2014; van Staa et al., 2002).

Genetic factors are important in PDB and several predisposing genetic variants have now been identified by a combination of linkage studies in families and genome-wide association studies (Albagha et al., 2010, 2011; Laurin et al., 2002; Ralston and Albagha, 2014; Scotto di Carlo et al., 2020; Vallet et al., 2015). Follow-up functional studies are essential to identify the gene(s) responsible for association with PDB at these loci and to define the mechanisms by which these genes regulate bone metabolism. For example, functional studies of the chromosome 10p13 susceptibility locus identified optineurin (*OPTN*) as the gene driving the association with PDB and elucidated the mechanism by which this gene regulates bone metabolism (Obaid et al., 2015; Wong et al., 2020). However, for many of these susceptibility loci, the genes responsible for driving the association with PDB are unknown. One of the predisposing loci identified for PDB by GWAS is located on chromosome 15q24. There are several genes at this locus (*LOXL1*, *PML*, *STOML1*, *GOLGA6A*, *ISLR* and *ISLR2*), but the strongest association is with a single nucleotide polymorphism (SNP), rs5742915, located within the coding region of the promyelocytic leukaemia gene (*PML*), which causes a phenylalanine to leucine amino acid substitution at codon 645 (p.Phe645Leu) (Albagha et al., 2011). The *PML* gene was so named as it was identified as a tumour suppressor gene that was disrupted in acute promyelocytic leukaemia, where it is fused to retinoic acid receptor alpha (*RARA*) gene as a result of the chromosomal translocation t(15;17) (Nisole et al., 2013; Salomoni and Pandolfi, 2002).

Previous studies have shown that PML is involved in various biological processes, including cell growth, senescence, apoptosis, protein degradation and antiviral response (Guan and Kao, 2015; Salomoni and Pandolfi, 2002). Until its discovery as a predisposing locus for PDB, PML had not been considered to play a role in bone metabolism but could be involved through its known effects on diverse bone-related signalling pathways such as NF-κB, TGF-β, IFN-γ, p38 and Wnt (El Bougrini et al., 2011; Lin et al., 2004; Shin et al., 2004; Shtutman et al., 2002; Wu et al., 2002). In this study, we have investigated the role of *PML* in bone cell function to gain an insight into the mechanisms by which PML affects bone metabolism and predisposes an individual to PDB.

## RESULTS

### PML is expressed in osteoclasts and osteoblasts

**Fig. 1.**
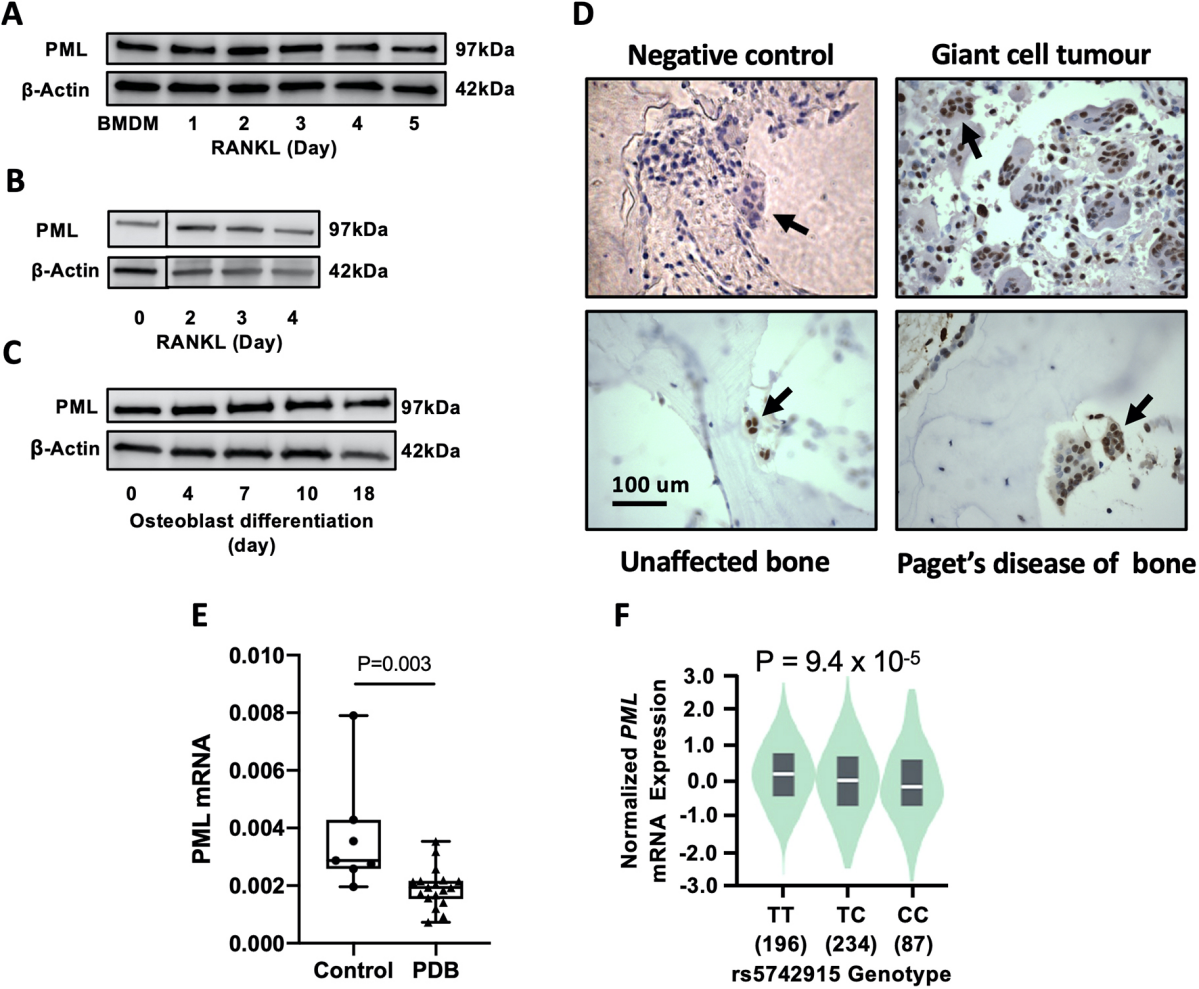
**PML expression in bone cells from mouse and human tissue.** (A) PML protein expression during osteoclast differentiation from mouse bone marrow-derived macrophages (BMDMs). (B) PML protein expression during osteoclast differentiation from RAW 264.7 cells. (C) PML protein expression during differentiation of mouse calvarial osteoblasts. (D) Detection of PML in human tissue sections using immunohistochemistry. Staining for PML appears dark brown. The negative control is a Pagetic bone sample processed without addition of the primary antibody; unaffected bone is from a patient with osteomyelitis. Arrows indicate osteoclasts in sections from the indicated bone conditions. Osteoclast staining for PML is observed in Pagetic bone, unaffected bone and giant cell tumour. PML staining is also evident in a row of osteoblasts at the top left of the section from Paget's disease of bone. Scale bar: 100 µm. (E) *PML* mRNA expression normalized for 18S rRNA in peripheral blood mononuclear cells (PBMCs) of PDB patients (*n*=18) and controls (*n*=7). (F) Normalized *PML* mRNA expression in relation to PML rs5742915 genotypes as extracted from the GTExPortal database (https://gtexportal.org/home/). The median values for the genotypes were: TT=+0.1140, TC=−0.0508 and CC=−0.2344. The western blot images shown in A-C are representative of three independent biological replicates. Data in E are presented as box and whisker plots showing the median (horizontal line), interquartile range (box) and range (whiskers). Data in F are presented as violin plots showing the median (white horizontal line) and interquartile range (black boxes). *P*-values are from linear regression analysis.

We found that PML protein was expressed in the mouse monocyte-macrophage cell line RAW 264.7, as well as in primary mouse bone marrow-derived macrophages (BMDMs). Expression of PML was detected at all stages during osteoclast differentiation following stimulation with RANKL (Fig. 1A,B). We also found that PML was expressed in mouse calvarial osteoblasts and during their differentiation to the stage of bone nodule formation (Fig. 1C).

To determine whether PML is expressed in human osteoclasts, we conducted immunostaining for PML protein in human osteoclasts from giant cell tumour of bone, and in bone sections from patients with PDB and from patients unaffected by PDB. This showed that PML protein was expressed in the nuclei of osteoclasts in all sections examined (Fig. 1D), as well as in osteoblasts that were visible in the PDB sample.

We also detected *PML* mRNA in peripheral blood mononuclear cells (PBMCs) and found that levels of expression were significantly lower in PBMCs from PDB patients (*n*=18) compared with unaffected controls (*n*=7) (*P*=0.01; Fig. 1E). Two out of the 18 patients with PDB were positive for the P392L mutation in *SQSTM1* but levels of *PML* mRNA expression in these subjects did not differ from the rest of the PDB cohort (Table S2). The number of PDB patients was too small to perform an expression quantitative trait locus (eQTL) analysis for PML in PBMCs but four samples of the PDB group were T/C heterozygotes at the rs5742915 SNP, which allowed us to investigate allele-specific gene expression. This showed that the mean±s.d. expression from the C allele was 19.0±3.8% lower than the expression from the T allele (*P*=0.0002), consistent with the hypothesis that allelic variants at rs5742915 are associated with reduced PML expression.

In order to confirm whether allelic variation at the rs5742915 SNP on 15q24 was an eQTL for PML, we scrutinised the GTEx portal (https://gtexportal.org/home/) and found that carriage of the C-allele at this SNP, which is associated with a 1.34-fold increased risk of PDB (Albagha et al., 2011), is also associated with reduced *PML* mRNA expression levels in skin cells (Fig. 1F). This indicates that reduced expression of PML increases the risk of PDB.

### Effect of PML overexpression on osteoclast differentiation in RAW264.7 cells

**Fig. 2.**
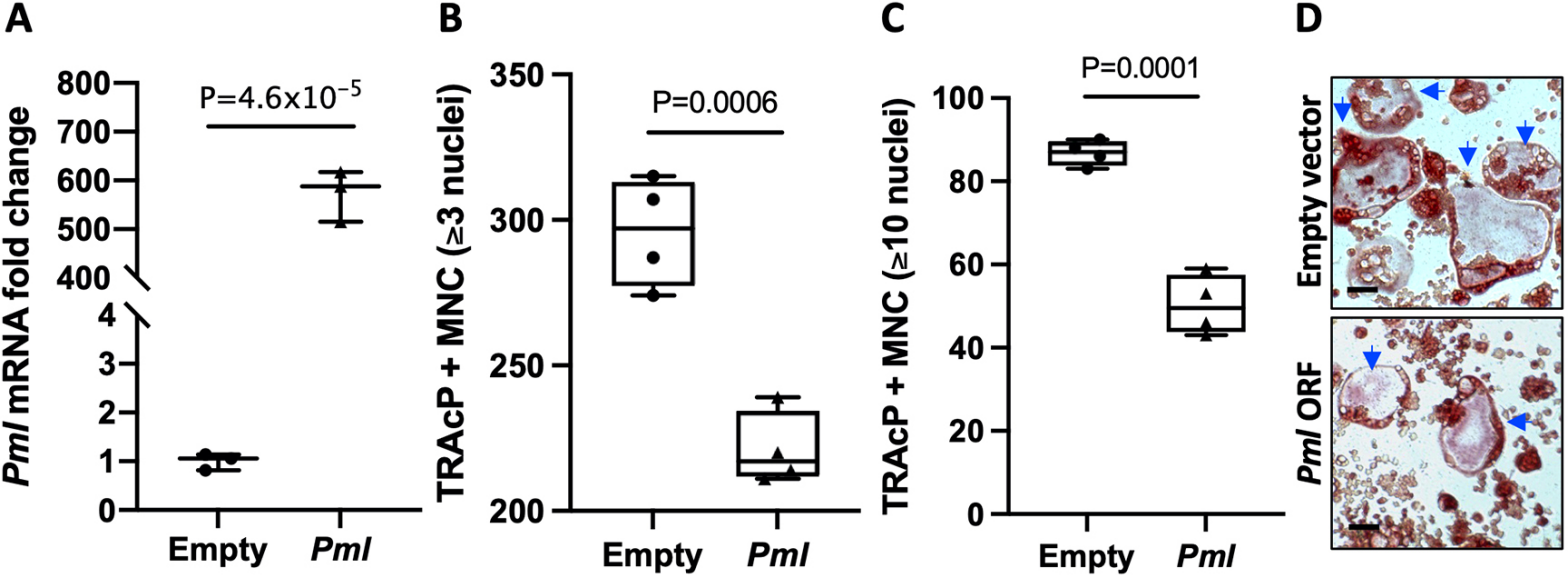
**Effect of altered *Pml* expression on osteoclast differentiation *in vitro*.** (A) *Pml* mRNA in RAW 264.7 cells overexpressing *Pml* compared with control cells (empty vector). mRNA levels were assayed by qRT-PCR and normalized for 18S rRNA, and are presented as fold increase in reference to control cells. (B) Number of tartrate-resistant acid phosphatase (TRAcP)^+^ multinucleated (MNC) osteoclasts (≥3 nuclei) generated from RAW 264.7 cells overexpressing *Pml* compared with control (empty vector). (C) Number of large osteoclasts (≥10 nuclei) generated from RAW 264.7 cells overexpressing *Pml* compared with control. (D) Representative images of TRAcP stained cultures showing reduction in size and number of osteoclasts (indicated by arrows) in *Pml*-overexpressing cells compared with control cells. Values are representative of three independent biological replicates. Data in A are presented as a scatter plot showing the median and range. Data in B and C are presented as box and whisker plots showing the median (horizontal line), interquartile range (box) and range (whiskers). *P*-values are from an unpaired Student *t*-test. Scale bars: 100 µm.

Given that PML expression was reduced in PDB patients, we studied the effect of altered PML expression on osteoclast differentiation in RAW 264.7 cells, a mouse monocyte-macrophage-like cell line that differentiates into osteoclast-like cells upon RANKL treatment. Overexpression of PML resulted in a significant reduction in the number and size of osteoclasts formed compared with empty vector (Fig. 2A-D).

### Effects of targeted inactivation of PML on bone metabolism

To investigate the effects of PML on skeletal phenotype *in vivo* we compared the characteristics of mice with targeted inactivation of PML (*Pml^−/−^*) (Wang et al., 1998) and wild-type littermates. Although the susceptibility alleles on the chromosome 15q24 locus predispose to PDB similarly in both men and women (Albagha et al., 2011), we decided to focus our analysis on male *Pml^−/−^* mice as PDB is about 40% more common in men than in women (van Staa et al., 2002).

The *Pml^−/^*^−^ and wild-type mice were phenotypically normal. There were no differences between genotypes in body weight, body habitus, dentition, gait or survival. In contrast to a previous study (Lunardi et al., 2011), we found no evidence to suggest that *Pml^−/−^* mice had increased susceptibility to infection.

### Osteoclast function in *Pml^−/−^* mice

**Fig. 3.**
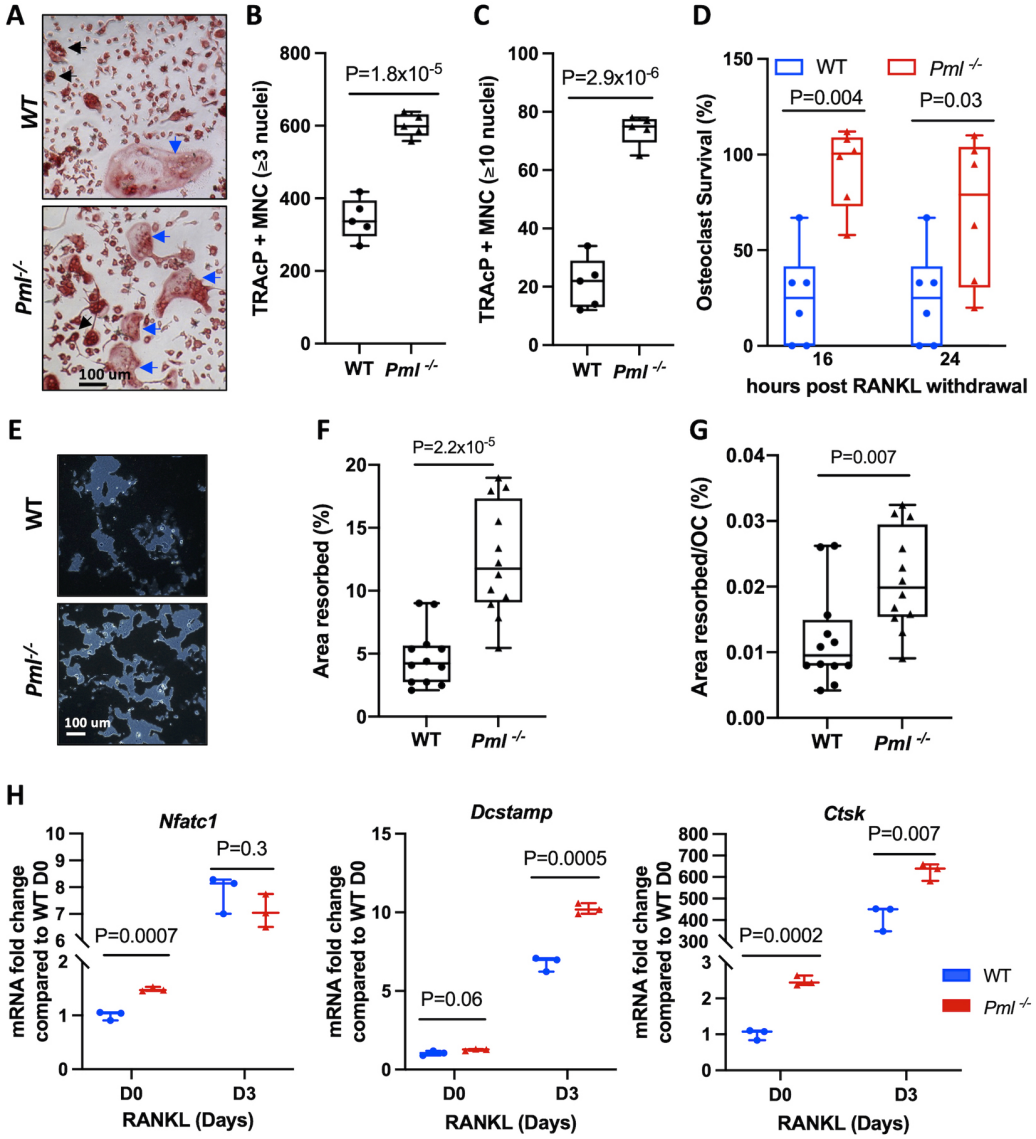
**Osteoclast cultures from *Pml^−/−^* mice.** (A) Representative images of TRAcP-stained cultures showing increase in the number and size of osteoclasts generated from *Pml*^−/−^ mice compared with wild type. Black and blue arrows indicate osteoclasts and large osteoclasts, respectively. (B) Number of TRAcP^+^ osteoclasts (≥3 nuclei) generated from BMDMs of *Pml*^−/−^ mice compared with wild type. (C) Number of TRAcP^+^ large osteoclasts (≥10 nuclei) generated from BMDMs of *Pml*^−/−^ mice compared with wild type. (D) Osteoclast survival following RANKL withdrawal in *Pml*^−/−^ mice compared with wild type at the indicated time points. (E) Representative images of modified Von-Kossa staining on an Osteo Assay plate showing greater resorption activity in osteoclast cultures from *Pml*^−/−^ mice compared with wild type. (F,G) Area resorbed by osteoclasts cultured on Osteo Assay plates from *Pml*^−/−^ mice compared with wild type presented as % of total well area (F) or as area resorbed per osteoclast (G). Values in B-D,F,G are representative of three independent biological replicates, each consists of at least six technical replicates. (H) Expression of *Nfatc1*, *Dcstamp* and *Ctsk* during osteoclast differentiation from BMDMs of *Pml*^−/−^ mice compared with wild type at the indicated time points after RANKL stimulation. Gene expression values were normalized for 18S rRNA and are presented as fold change in relation to wild-type cultures before RANKL stimulation (D0). Values in H are combined from three independent biological replicates. Data in B-D,F,G are presented as box and whisker plots showing the median (horizontal line), interquartile range (box) and range (whiskers). Data in H are presented as a scatter plot showing the median and range. *P*-values are from a two-tailed unpaired Student's *t*-test. Scale bars: 100 µm.

Osteoclasts generated *in vitro* from bone marrow-derived macrophages from *Pml^−/−^* mice were significantly greater in number and larger in size when compared with those from wild-type littermates (Fig. 3A-C). Survival of osteoclasts from *Pml^−/−^* mice was also significantly prolonged after RANKL withdrawal when compared with wild type (Fig. 3D). Furthermore, osteoclasts generated from *Pml^−/−^* mice showed higher resorption activity compared with those from wild type (Fig. 3E-G). Taken together, these data indicate that absence of *Pml* results in a significant increase in osteoclast formation, activity and survival.

In order to gain insight into the molecular mechanisms of osteoclast activation in *Pml*^−/−^ mice, we compared the expression of key osteoclast-related genes *Nfatc1*, *Dcstamp* and *Ctsk* in *Pml*^−/−^ and wild type during RANKL-induced osteoclast differentiation. These genes were chosen as being representative of a range of genes that are activated during osteoclast differentiation (Asagiri and Takayanagi, 2007). This revealed higher levels of *Nfatc1* expression in osteoclast precursors from *Pml^−/−^* mice compared with wild type WT (Fig. 3H). We also observed significant increase in *Dcstamp* expression during osteoclast differentiation in both wild type and *Pml^−/−^* but the expression of this gene was significantly higher in *Pml^−/−^* compared with wild type during later stages of osteoclast differentiation. Additionally, the expression of the osteoclast marker gene cathepsin K (*Ctsk*) was significantly higher in *Pml^−/−^* compared with wild type in osteoclast precursors, as well as during their differentiation into osteoclasts (Fig. 3H).

### PML regulates the inhibitory effect of IFN-γ on osteoclast differentiation

**Fig. 4.**
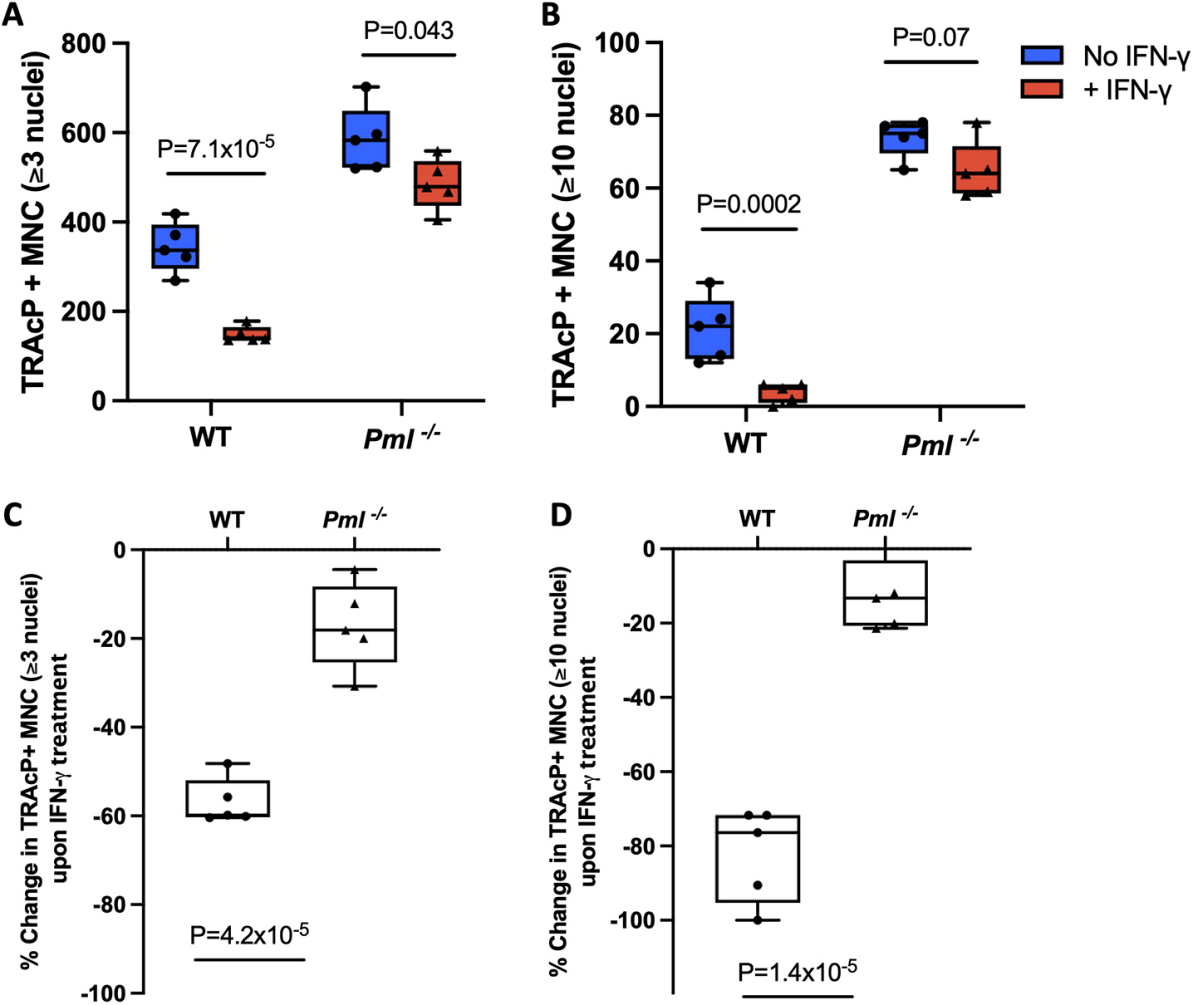
**Effect of IFN-γ on osteoclast differentiation in *Pml*^−/−^ mice.** (A) Number of TRAcP^+^ multinucleated (MNC) osteoclasts (≥3 nuclei) generated from BMDMs of *Pml^−/−^* mice in the presence or absence of IFN-γ compared with wild type. (B) Number of large osteoclasts (≥10 nuclei) generated from BMDMs of *Pml^−/−^* mice in the presence or absence of IFN-γ compared with wild type. BMDMs were treated with IFN-γ (5 ng/ml or 24 h) followed by stimulation with M-CSF and RANKL until osteoclasts were formed. (C) Percentage reduction in osteoclast numbers (≥3 nuclei) upon treatment with IFN-γ in *Pml^−/−^* mice compared with wild type. (D) Percentage reduction in large osteoclast numbers (≥10 nuclei) upon treatment with IFN-γ in *Pml^−/−^* mice compared with wild type. The percentage change data in C and D are derived from the data in A and B, respectively. The values shown are representative of three independent biological replicates, each consisting of at least five technical replicates. Box and whisker plots show median (horizontal line), interquartile range (box) and range (whiskers). *P*-values are from a two-tailed unpaired Student's *t*-test.

Interferon gamma (IFN-γ) is a critical regulator of osteoclast differentiation (Takayanagi et al., 2000) and previous studies have shown that PML positively regulates IFN-γ signalling (El Bougrini et al., 2011). In view of this, we investigated the effect of IFN-γ on osteoclasts differentiated from bone marrow-derived macrophages in *Pml^−/−^* mice. These studies showed that, while IFN-γ inhibited osteoclast differentiation in wild-type (*P*=7.1×10^−5^) and *Pml^−/−^* (*P*=0.043) mice. The inhibitory effect of IFN-γ was significantly blunted in *Pml^−/−^* mice compared with wild type, particularly with regard to large osteoclasts (≥10 nuclei) (Fig. 4A,B). Treatment of cultures with IFN-γ resulted in a significant decrease in the number of osteoclasts from wild type (56.8%) compared with *Pml^−/−^* (17.1%; *P*=4.2×10^−5^) (Fig. 4C). Similarly, the reduction in the number of large osteoclasts upon treatment with IFN-γ was significantly higher in cultures from wild type (82.1%) compared with those from *Pml^−/−^* (12.2%; *P*=1.4×10^−5^, Fig. 4D). These observations indicate that the inhibitory effect of IFN-γ on osteoclast generation is partly dependent on PML.

### Osteoblast function in *Pml^−/−^* mice

**Fig. 5.**
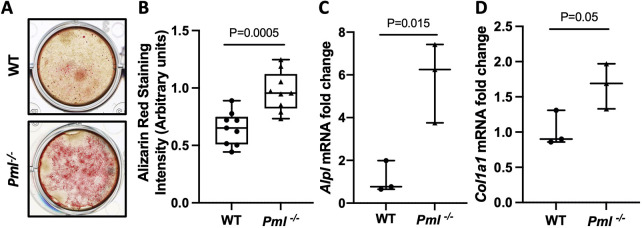
**Osteoblast cultures from *Pml^−/−^* mice.** (A) Representative images of mineralized nodules stained with Alizarin Red in calvarial osteoblasts after 18 days in culture from *Pml*^−/−^ compared with wild-type mice. (B) Bone nodule formation in *Pml*^−/−^ mice compared with wild-type osteoblast cultures at day 18, as determined by Alizarin Red staining, which was measured by absorbance at 562 nm; values are corrected for the number of viable cells. (C,D) Expression of alkaline phosphatase (*Alpl*) mRNA (C) and collagen 1 α 1 (*Col1a1*) mRNA (D) in calvarial osteoblasts from *Pml*^−/−^ mice compared with wild type at day 1 of culture. Gene expression values were normalized for 18S rRNA and are presented as fold change in relation to wild-type cultures. Data are representative (A,B) or combined (C,D) from three independent biological replicates. Data in B are presented as a box and whisker plot showing the median (horizontal line), interquartile range (box) and range (whiskers). Data in C,D are presented as scatter plots showing the median and range. *P*-values are from a two-tailed unpaired Student's *t*-test.

We investigated the role of PML in osteoblast function by conducting mineralising bone nodule assays in calvarial osteoblasts derived from *Pml^−/−^* and wild-type mice after 18 days culture in osteogenic medium. This showed that bone nodule formation was significantly greater in *Pml^−/−^* mice compared with wild type (Fig. 5A,B). Additionally, the expression of the osteoblast marker gene alkaline phosphatase (*Alpl*) was significantly higher in proliferating osteoblasts (day 1 of culture) from *Pml^−/−^* mice compared with wild type (*P*=0.015; Fig. 5C). In addition, expression of *Col1a1* was higher in osteoblasts from *Pml^−/−^* compared with wild type but this was of borderline significance (*P*=0.05; Fig. 5D).

### Bone turnover in *Pml^−/−^* mice

To investigate the effect of PML inactivation on bone turnover *in vivo*, we performed bone histomorphometry in *Pml^−/−^* and wild-type mice. Static bone histomorphometry parameters showed increased bone resorption parameters in male *Pml^−/−^* mice, as shown in Table 1 and Fig. S1. Osteoclast surface/bone surface (Oc.S/BS), osteoclast number/bone surface (N.Oc/BS) and osteoclast number/tissue volume (N.Oc/TV) were approximately 40-50% higher in *Pml^−/−^* mice compared with wild type. Dynamic bone histomorphometry showed significant 15% increase in the mineral apposition rate (MAR) in *Pml^−/−^* compared with wild-type mice (Table 1 and Fig. S1). There was also a trend for higher bone formation rate per bone surface (BFR/BS) in *Pml^−/−^* mice compared with wild type but this did not reach statistical significance (*P*=0.09; Table 1).

**Table 1.**
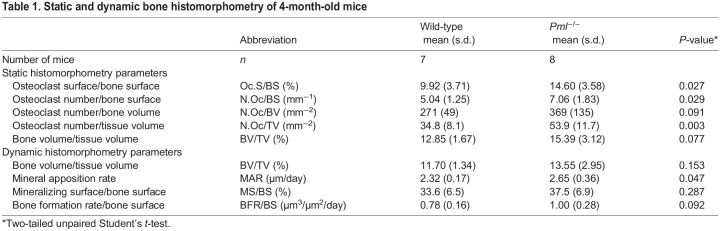
Static and dynamic bone histomorphometry of 4-month-old mice

### Bone volume and structure in *Pml^−/−^* mice

We analysed bone volume and structure of *Pml^−/−^* and wild-type mice using microcomputed tomography (µCT). We found no significant differences in trabecular or cortical bone parameters in the hind limbs of male *Pml^−/−^* compared with wild type at 4 months of age (Tables 2 and 3). We also searched for evidence of Pagetic-like lytic lesions in male *Pml^−/−^* and wild-type mice at 4 months but none was detected (data not shown). We went on to study aged *Pml*^−/−^ and wild type at 14 months of age. We found no difference in BV/TV, trabecular separation or number. However, we observed a significant 16% reduction in trabecular tissue volume (TV) in *Pml^−/−^* mice compared with wild type (Table 2). Likewise, TV was also significantly lower in cortical bone of 14-month-old *Pml^−/−^* mice compared with wild type, although cortical thickness was not affected (Table 3). However, both the periosteal and endosteal perimeters were decreased by 7% and 10%, respectively, in *Pml^−/−^* mice compared with wild type, which, together with the decreased tissue volume, indicates a reduction in bone size (Table 3). This resulted in a significant 20% reduction in moment of inertia along all axes in *Pml^−/−^* compared with wild type. However, the µCT scans of the hind limbs of the 14-month-old mice did not reveal any evidence of Pagetic-like bone lesions. Table S2 provides a breakdown of number of mice analysed in each age group.

**Table 2.**
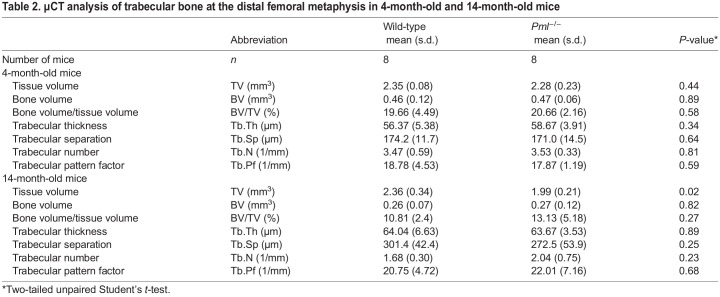
µCT analysis of trabecular bone at the distal femoral metaphysis in 4-month-old and 14-month-old mice

**Table 3.**
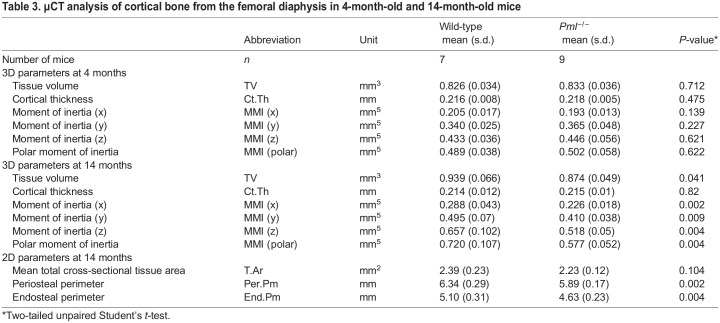
µCT analysis of cortical bone from the femoral diaphysis in 4-month-old and 14-month-old mice

## DISCUSSION

The chromosome 15q24 locus was detected as a susceptibility locus for PDB by an extended genome wide association study (Albagha et al., 2011; Ralston and Albagha, 2014). Although there are multiple candidate genes at this locus, the strongest association was with rs5742915, which is located within the coding region of *PML*, causing and amino acid change (p.Phe645Leu). Here, we have investigated the role of PML in bone metabolism using *Pml* knockout mice and identified a new role for this gene in regulating bone cell function.

We found that PML was expressed in both osteoclasts and osteoblasts, as well as in RAW 264.7 cells. We also gained robust evidence to show that PML acts as a negative regulator of osteoclast differentiation and function. In RAW 264.7 cells, we found that overexpression of *Pml* suppressed osteoclast differentiation. The negative regulatory role of PML on osteoclast was confirmed by studies of osteoclast function in mice with targeted inactivation of *Pml*. We found evidence of increased osteoclast differentiation, increased osteoclast size and multinuclearity, increased survival and increased resorptive activity in cells derived from *Pml^−/−^* mice compared with wild-type littermates. The increased expression of *Nfatc1* in osteoclast precursors (BMDMs) from *Pml*^−/−^ mice appears to promote their differentiation down the route of osteoclast lineage. The increased expression of *Dcstamp*, an essential regulator of osteoclast fusion (Yagi et al., 2005), could account for larger osteoclasts in cultures from *Pml*^−/−^ mice. Similarly, the increased expression of *Ctsk*, a proteinase involved in bone resorption, could explain the enhanced resorption activity of osteoclast in *Pml^−/−^* mice.

It has long been established that IFN-γ derived from T-cells abrogates RANKL-induced osteoclast formation by inhibiting tumour necrosis factor receptor-associated factor 6 (TRAF6) (Takayanagi et al., 2000). IFN-γ also modulates the expression of CTSK in pre-osteoclastic cells (Pang et al., 2005), as well as in mature osteoclasts (Kamolmatyakul et al., 2001). Additionally, the interferon regulatory factor 8 (IRF8) suppresses osteoclast differentiation by inhibiting NFATc1 (Zhao et al., 2009). Studies have also shown that PML expression is upregulated by IFN-γ in mouse peritoneal macrophages (Dror et al., 2007). Here, we report for the first time that the inhibitory effect of IFN-γ on osteoclast differentiation is partly dependent on PML as bone marrow cultures from *Pml^−/−^* mice were relatively resistant to the suppressive effects of IFN-γ on osteoclast formation when compared with wild type. Considering the above findings, PML could be implicated in osteoclast signalling by modulating the IFN-γ pathways in association with direct or indirect regulation of osteoclast-related genes such as NFATc1, DCSTAMP and CTSK. In *Pml^−/−^* mice, osteoclasts are already primed for increased formation, differentiation, fusion and activity due to upregulation of these factors in absence of PML, thereby rendering negative regulators less effective in suppressing osteoclastogenesis. However, IFN-γ signalling in bone is complex as studies have also shown that, in addition to its direct inhibitory effect on osteoclasts (Takayanagi et al., 2000), IFN-γ has an indirect pro-osteoclastogenic effect *in vivo* through activation of T cells and stimulation of RANKL and TNF-α secretion from activated T cells (Gao et al., 2007). Similarly, IFN-γ has variable effects on osteoblasts; a pro-osteoblastic effect has been reported (Duque et al., 2011, 2009; Rifas, 2006), but Xiao et al. have shown an indirect inhibitory effect on osteoblasts (Xiao et al., 2004). Therefore, further detailed studies are needed to elucidate the crosstalk between PML and IFN-γ, and how they influence bone cell function.

There are other potential mechanisms by which PML could influence osteoclast and osteoblast function. One is through its effect as an inhibitor of the p38 MAPK pathway (Shin et al., 2004). This pathway plays an important role in bone metabolism by stimulating osteoclast formation, maturation and bone resorption, as well as by regulating osteoblast differentiation, extracellular matrix deposition and bone mineralisation (Greenblatt et al., 2010; Thouverey and Caverzasio, 2015). It is therefore possible that a reduction in *Pml* expression could increase p38 MAPK signalling, thereby contributing to the high bone turnover seen in the *Pml^−/−^* mice and in humans with PDB. Another possible effect of PML on bone cell function would be through an autophagy-mediated mechanism, as PML has been shown to interact with both p62 and the autophagy effector protein LC3 (Li et al., 2020). However, further work would be required to determine whether crosstalk between PML and p62 plays a role in regulating bone cell function and whether reduced levels of PML may affect this process.

We also found that PML negatively regulates osteoblast function, as reflected by the increase in bone nodule formation on osteoblast cultures from *Pml^−/−^* mice compared with wild type. This was accompanied by increases in key osteoblast markers, such as alkaline phosphatase (*Alpl*) and *Col1a1* in *Pml*^−/−^ osteoblasts compared with wild type. These changes in early stages of osteoblast differentiation could lead to increased osteoblast formation, differentiation and activity, thereby resulting in increased bone formation. There have been very few previous studies in the effects of PML on osteoblast function, but in a study by Sun et al. (Sun et al., 2013), it was reported that overexpression of PML in human mesenchymal stem cells inhibited cell proliferation by causing apoptosis but also increased alkaline phosphatase activity. These findings differ somewhat from the present study where we found that *Pml^−/−^* osteoblasts had an increased propensity to form bone nodules *in vitro* and that *Pml^−/−^* mice had evidence of increased bone formation. We speculate that these differences may be accounted for by the differences in experimental design, by the use of stem cells in the previous study and the use of clavarial osteoblast cells in this study.

Skeletal phenotyping of young adult male mice (4 months old) using µCT revealed no significant differences in trabecular or cortical bone volume or structure between *Pml^−/−^* mice and wild-type littermates. Although the histomorphometric studies showed evidence of increased bone resorption and bone formation in *Pml^−/−^* mice, it seems that the tight coupling between these processes resulted in no overall change in bone mass or bone structure in the different genotype groups. Analysis of aged male mice (14 months old) using µCT similarly revealed no significant differences in trabecular bone volume or structure between *Pml*^−/−^ mice and wild type but there was a significant reduction in bone size in *Pml*^−/−^ mice. This was evident from lower trabecular and cortical tissue volume and the decreased periosteal and endosteal perimeters in *Pml^−/−^* mice, which is accompanied by reduction in moment of inertia (MMI) at all axes. MMI is a geometry-dependent parameter that predicts resistance to bending (or torsion in the case of the polar moment of inertia) of a structure. Bones with a smaller cross-sectional area, but the same cortical thickness will have a lower MMI. During long bone growth, bone is shaped by resorption on the outside (periosteum) and formation on the inside (endosteum). Therefore, increased osteoclastic bone resorption could lead to excess resorption on the outside, compensated for by increased endosteal bone formation, leading to a decrease in bone perimeter.

Despite the overall increase in bone turnover, we observed no evidence of focal bone lesions in these mice as occurs in human PDB. This indicates that deletion of PML is not sufficient to cause PDB-like bone lesions in mice, contrasting with mouse models of two other PDB-susceptibility genes: *SQSTM1* P394L knock-in mice (Daroszewska et al., 2018, 2011) and mice with deletion or loss of function of the *Optn* gene (Obaid et al., 2015; Wong et al., 2020), both of which develop PDB-like bone lesions with increasing age. This difference may be due to the fact that the effect size of the rs5742915 susceptibility allele at the PML locus in predisposing to PDB is modest (∼1.34 fold) when compared with the susceptibility alleles at the *SQSMT1* and *OPTN* loci, which are of considerably larger effect size, resulting in Mendelian forms of PDB (Hocking et al., 2002; Laurin et al., 2002; Obaid et al., 2015).

Although we have no reason to suspect that the effects of PML on bone metabolism differ in males and females, a limitation of the preclinical studies described here was that the skeletal phenotyping was restricted to male mice. We chose to study males because PDB is more common in men but acknowledge that further studies to investigate bone metabolism in female *Pml^−/−^* mice would be of interest.

In contrast to a previous report (Lunardi et al., 2011), we did not observe any difference in survival between *Pml^−/−^* mice and littermates, nor did we observe increased susceptibility to infections. The reasons for this are not entirely clear but are likely to be due to differences in animal husbandry as the increased risk of infections noted by Lunardi was observed only when the mice were kept in non-pathogen-free conditions or were directly challenged with micro-organisms. In this study, however, the mice were kept in specific-pathogen-free conditions.

In summary, our findings are consistent with a model whereby genetic variations at the 15q24 locus predispose individuals to PDB by reducing expression of PML, which stimulates osteoclastic bone resorption and bone formation. Although the PML variant associated with increased risk of PDB (rs5742915_T>C) results in an amino acid change, it is not predicted to be pathogenic. However, further studies are warranted to determine how this variant regulates the expression of PML and to determine whether it does so directly or through linkage disequilibrium with another nearby functional variant.

## MATERIALS AND METHODS

### Reagents and materials

The following media were used: Minimal Essential Medium Eagle (αMEM, Sigma), Dulbecco's modified Eagle medium (DMEM, Sigma), foetal calf serum (HyClone Laboratories) and L-Glutamine (Invitrogen). Complete media were supplemented with 10% foetal calf serum (FCS), 2 mM L-glutamine, 100 U/ml penicillin and 100 µg/ml streptomycin.

The following antibodies were used: rabbit anti-PML (sc-5621; 1:500; SantaCruz Biotechnology), rabbit anti-actin IgG (AA20-33; 1:1000; Sigma). The cytokines murine M-CSF (Prospec Bio), human recombinant RANK-L (R&D Systems) and IFN-γ (Thermo Fisher Scientific) were used.

Other reagents, kits and cell lines used were: penicillin (Invitrogen), streptomycin (Invitrogen), geneticin (G418; Thermo Fisher Scientific), cell dissociation buffer (Thermo Fisher Scientific), jetPEI-Macrophage (Polyplus transfection), Alamar Blue reagent (Invitrogen), GenElute Mammalian Total RNA kit (Sigma), RNeasy kit and DNeasy blood and tissue kit (Qiagen), qScript cDNA SuperMix kit (QuantaBioscience), SensiFAST Probe No-ROX kit (Bioline), Ambion Ribopure Blood RNA isolation kit (Thermo Fisher Scientific), Osteo Assay plates (Corning) and RAW 264.7 (ATCC). All cultures were performed in standard conditions of 5% CO_2_ and 37°C in a humidified atmosphere.

### Mice

The *Pml^−/−^* mice were obtained from the National Cancer Institute, USA and were generated as previously described (Wang et al., 1998). Briefly, *Pml* was disrupted in the mouse germ line and knockout generated by deleting part of exon 2 (94 bp) which encodes the RING finger domain. Complete absence of *Pml* in these mice was verified by southern and northern blotting, as well as immunofluorescence staining (Wang et al., 1998). The animals bred and maintained for the study were genotyped to confirm their status as per the protocols specified by the National Cancer Institute's mouse repository. The *Pml^−/−^* and wild-type mice used in the experiments were littermates on a C57BL/6 background. The skeletal phenotyping experiments described were conducted on male *Pml^−/−^* mice. The mice were housed in a standard animal facility (specific-pathogen free) with free access to food (pelleted RM1; SDS diets) and water. All experiments on mice were performed according to institutional, national and European animal regulations.

### μCT

Mouse hindlimbs were imaged by µCT using a Skyscan 1272 µCT scanner (Bruker) as described previously (van ‘t Hof and Dall'Ara, 2019). Briefly, hind limbs of mice were dissected free of most soft tissue, fixed in 4% buffered formaldehyde for 24 h, stored in 70% ethanol and scanned at a resolution of 5 µm (60 kV, 150 µA, rotation step size 0.3°, 0.5 mm aluminium filter). Image reconstruction was performed using the Skyscan NRecon package. Skyscan Dataviewer software was used to orientate the image stacks and create subvolumes for subsequent image analysis. The reconstructed µCT images were also subjected to 3D analyses using Skyscan Dataviewer software to screen for bone lesions and CT Vol software was used in addition to generate 3D model images of bones.

Trabecular bone was analysed in a stack of 200 slices starting 100 µm from the distal femoral growth plate. Cortical parameters were measured in 100 slices at the midshaft of the femur. Trabecular and cortical bone parameters were measured in Skyscan CTAn software using a fixed threshold and automated separation of cortical and trabecular bone using a custom macro.

### Bone histomorphometry

Bone histomorphometry was performed at the distal femoral metaphysis essentially as described in van ‘t Hof et al. (2017). Briefly, mice received intraperitoneal calcein injections (2 mg/ml, 150 µl) 5 days and 2 days before culling. Hind limbs were fixed for 24 h in 4% buffered formalin and embedded in methyl methacrylate (MMA). Sections (5 µm) were cut using a tungsten steel knife on a Leica motorized rotary microtome, and stained for tartrate-resistant acid phosphatase (TRAcP) to visualise osteoclasts and counterstained with Aniline Blue. For analysis of calcein double labelling, sections were counterstained with Calcein Blue. The only difference from the methods described by van ‘t Hof et al. (2017) is that, for the TRAcP stain, the slides were coverslipped using 80% glycerol rather than Apathy's serum. Sections were imaged using a Zeiss Axio Scan.Z1 slide scanner and histomorphometry performed using the TrapHisto and CalceinHisto open-source image analysis programs (van ‘t Hof et al., 2017). Three sections at least 100 µm apart were analysed for each sample, and the area analysed extended 1.75 mm proximal to the distal femoral growth plate.

### Overexpression of Pml in RAW 264.7 cells

The RAW 264.7 cells were cultured at a density of 10^4^ cells/well in 96-well plates in complete DMEM for 4 days with RANKL (100 ng/ml) until osteoclasts were formed. For the overexpression experiments, the RAW 264.7 cells were transfected with GenEZ Mouse *Pml* ORF expression plasmid DNA (OMu22116-Genscript; pcDNA 3.1 plasmid containing mouse *Pml* ORF) or empty vector DNA (pcDNA3.1 alone) using jetPEI according to the manufacturer's protocol. The cells were transiently selected for 3-4 days using 500 μg/ml geneticin. Overexpression was confirmed by qRT-PCR. Cells overexpressing *Pml* or control cells were seeded in 96-well plate (10,000/well) in supplemented DMEM (150 μl/well) and stimulated with RANKL (100 ng/ml) for 4 days. The cultures were fixed with 4% formaldehyde in PBS. Multinucleated osteoclast-like cells were visualized using TRAcP staining and counted in each well. Osteoclasts were defined as TRAcP-positive cells with three or more nuclei and those containing more than 10 nuclei were designated as large osteoclasts. Each experiment was repeated at least three times with four to six technical replicate wells per experiment.

### Primary osteoclast cultures

Bone marrow cells were isolated from the tibiae and femurs of *Pml^−/−^* and wild-type littermates from 3- to 4-month-old mice and cultured in complete αMEM in the presence of M-CSF (100 ng/ml) for 2 days to generate bone marrow-derived macrophages (BMDMs). Adherent cells were re-seeded at a density of 10^4^ cells/well in 96-well plates, stimulated with M-CSF (25 ng/ml) and RANKL (100 ng/ml) for 4-5 days until osteoclasts were formed. The cultures were then fixed with 4% formaldehyde in PBS. Multinucleated osteoclasts were visualized using tartrate-resistant acid phosphatase (TRAcP) staining and counted in each well. Osteoclasts were defined as TRAcP-positive cells with three or more nuclei; those containing more than 10 nuclei were designated as large osteoclasts. For IFN-γ experiments, BMDMs were treated with IFN-γ (5 ng/ml) for 24 h followed by treatment with M-CSF and RANKL to generate osteoclasts. For survival experiments, osteoclasts were generated from BMDMs as described above. RANKL was then withdrawn from culture medium while retaining all other reagents. Plates were fixed at the indicated time points post-RANKL withdrawal, stained with TRAcP and osteoclasts counted. Each experiment was repeated using bone marrow cells from at least three different mice with four to six technical replicate wells per experiment.

### Bone resorption assay

The bone resorption activity of osteoclasts was determined using Osteo Assay surface 24-well plates (Corning). BMDMs were plated in Osteo Assay plates (50,000 cells/well in 500 μl/well supplemented αMEM) and differentiated into osteoclasts, as described above in osteoclast cultures. Wells were then treated with 2% sodium hypochlorite solution for 5 min, washed with distilled water and air-dried. For modified Von-Kossa staining, plates were treated, away from light, with 5% (w/v) silver nitrate solution for 30 min and then rinsed for 5 min with distilled water. Wells were then incubated in 5% (w/v) sodium carbonate in formalin solution for 5 min and then washed twice with distilled water followed by drying at 50°C for 1 h. Plates were then imaged with a Zeiss inverted microscope and resorption areas in each well were analysed using ImageJ software (Schneider et al., 2012). Each experiment was repeated using bone marrow cells from at least three different mice with four to six technical replicate wells per experiment.

### Primary osteoblast cultures

Osteoblasts were isolated from the calvarial bones of 2- to 4-day-old mice by sequential collagenase/EDTA digestion and cultured in 75 cm^2^ tissue culture flasks in complete αMEM. On reaching confluence after 2-3 days, cells were detached with trypsin, re-plated in 12-well plates at a density of 10^5^ cells/well and cultured in osteogenic medium (αMEM supplemented with 10% FCS, 50 µg/ml vitamin C and 3 mM β-glycerol phosphate). The medium was replaced three times per week and cultures were continued for up to 18 days until mineralized bone nodules formed. The cells were then fixed in 70% ethanol, washed in PBS, stained with Alizarin Red and left to dry overnight. Bone nodule formation was quantified by de-staining the cultures in 10% (wt/vol) cetylpyridinium chloride (Sigma Aldrich) and dissolving the stain in 10 mM sodium phosphate (pH 7.0). The absorbance of the extracted stain was then measured by a Bio-Tek Synergy HT plate reader at 562 nm and compared with an Alizarin Red standard curve. Alizarin Red values were corrected for the number of viable cells, as determined by the Alamar Blue assay. Each experiment was repeated using osteoblast-like cells from at least three different mice with four to six technical replicate wells per experiment.

### Immunohistochemistry

PML protein expression was detected in human osteoclasts by immunohistochemistry (IHC) of formalin-fixed paraffin embedded (FFPE) sections using PML antibody (sc-5621; 1:200) following standard protocols. The immunohistochemistry experiments included a negative control in which the bone section was processed without the addition of primary antibody.

### Immunoblotting

Cells were lysed using radioimmunoprecipitation assay (RIPA) buffer, centrifuged and protein concentration was measured using the Pierce protein assay. Proteins were loaded on Mini Protean TGX Precast gel electrophoresis system and electroblotted onto BioRad Mini PVDF membranes using Transblot Turbo transfer system. Membranes were blocked with 5% (w/v) non-fat milk in Tris-buffered saline (Thermo Scientific Pierce) with Tween-20 [TBST: 50 mM Tris, 150 mM NaCl and 0.1% (v/v) Tween-20] and probed with relevant primary antibody. After washing with TBST, membranes were incubated with anti-rabbit horseradish peroxidase-conjugated secondary antibody (1:5000, Cell Signaling) washed and visualized using Clarity Western ECL kit (BioRad) on a Licor Odyssey imager.

### Quantitative real-time PCR (qRT-PCR)

Total RNA was isolated using GenElute Mammalian Total RNA Kit and RNA was quantified using the Nanodrop 1000 Spectrophotometer. Complementary DNA was generated by RT-PCR using the qScript cDNA SuperMix kit following the manufacturer's instructions. Primers and fluorescently labelled probes were designed using the Primer 3 and the Roche Diagnostics website (Roche). Table S1 describes primer sequences and other details for target genes analysed by qPCR. Real-time PCR was performed on diluted cDNA using SensiFAST Probe No-ROX kit on a Chromo 4TM Detector/Bio RAD CFX Connect system and analysed using the Opticon MonitorTM software version 3.1 or Bio RAD CFX Manager V1.0. Samples were normalized to 18S rRNA expression. 18S cDNA was amplified with the VIC-labelled predesigned probe-primer combination from Applied Biosystems (4319413E) allowing two-channel detection of one cDNA. rs5742915 allele-specific expression of PML was performed using fluorescently labelled TaqMan probes (Applied Biosystems, 4351379) by following the manufacturer's protocol.

### Study subjects

We analysed PML expression in sections of human bone biopsies obtained from the NHS Lothian Bioresource. These included patients with PDB, giant cell tumour of bone and osteomyelitis in which there was histological evidence of active bone remodelling in the absence of PDB. We analysed PML expression in peripheral blood mononuclear cells obtained from patients with PDB attending routine outpatient clinics and unaffected controls. The PBMCs were isolated by density gradient centrifugation, RNA extracted and cDNA prepared according to standard techniques. Expression of *PML* was analysed by quantitative RT-PCR as described above.

### Ethics

Use of FFPE tissue samples was approved and agreed by the NHS Lothian Bioresource with ethical consent. Peripheral blood cells were obtained following written informed consent of all study subjects and approved by the local research ethics committee. All animal experiments were approved by the Animal Welfare and Ethical Review Body of the University of Edinburgh and were conducted in accordance with the UK Animals (Scientific Procedures) Act 1986 (project licence P2B36BCB).

### Statistical analyses

Analysis was performed using SPSS (IBM) and Prism ver 8.4 (GraphPad). Box and whiskers plot show the interquartile range (boxes), median (line inside boxes) and range (whiskers). A two-tailed unpaired Student's *t*-test was used for comparisons between two groups. *P*<0.05 was considered to indicate statistical significance. *PML* mRNA expression data in PDB patients and controls were analysed using linear regression with adjustment for age and gender. Experiments were performed as independent replicates and data presented as indicated in figure legends.

## Supplementary Material

Supplementary information
