# Supplementary material for: The Paget's disease of bone risk gene PML is a negative regulator of osteoclast differentiation and bone resorption
Source: Dis Model Mech. 2022 Apr 26;15(4):dmm049318. doi: 10.1242/dmm.049318 (PMC9066519; doi:10.1242/dmm.049318)
Supplement: Supplementary information [file dmm-15-049318-s1.pdf]

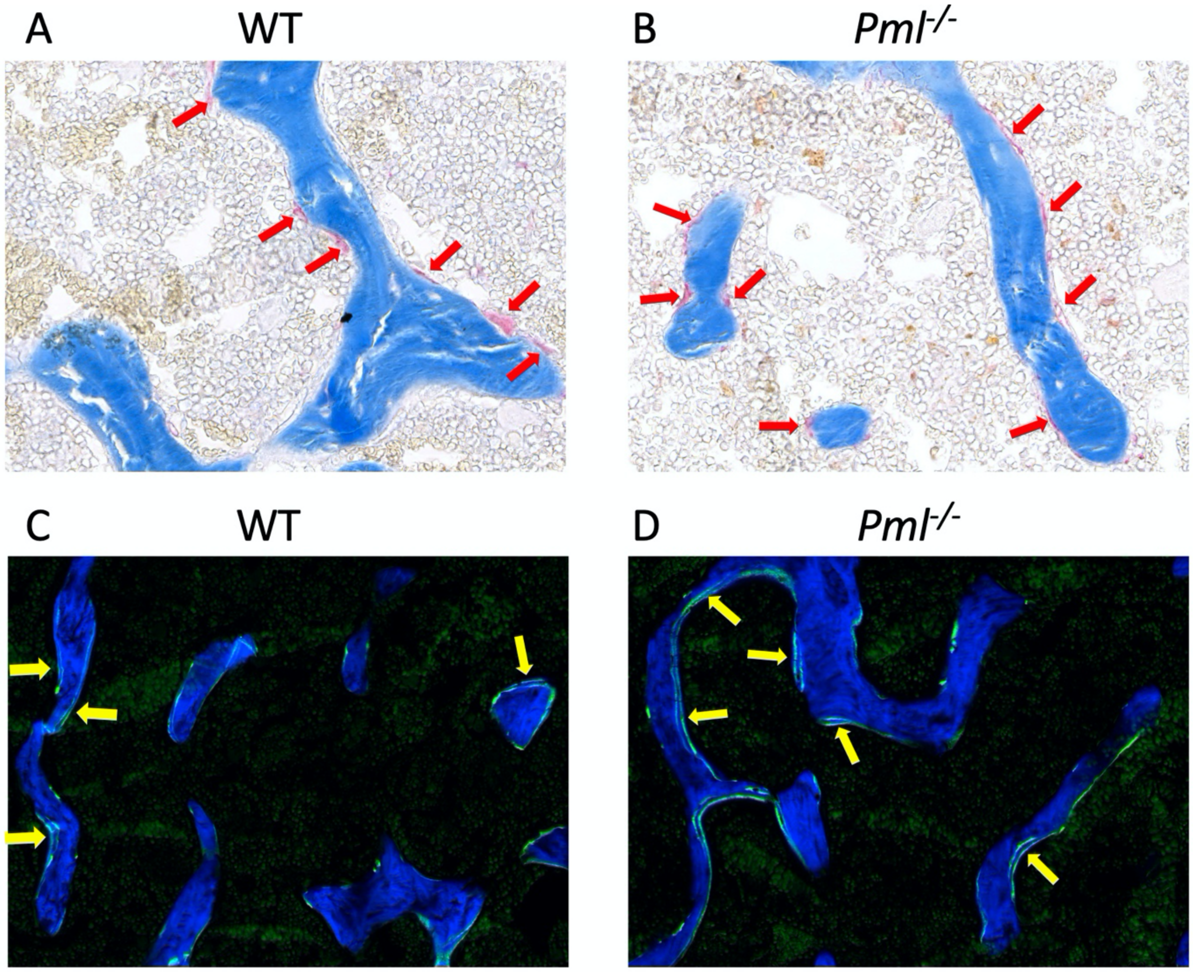

Fig. S1. Histomorphometrical analysis of trabecular bone from *Pml*$^{-/-}$ mice and WT controls. Representative images of bone sections from the distal femoral metaphysis stained with TRAcP (A,B) or calcein double labelling (C,D). Red arrows indicate TRAcP positive osteoclasts and yellow arrows show calcein double labelled surfaces.

Disease Models & Mechanisms • Supplementary information

Table S1. Primers and probes for qRT-PCR.

Target Gene	Primers (5' - 3') (F:Forward; R:Reverse)	Probe Number	Amplicon (bp)	Species
PML	F: ccgagacccccctctgaag<br>R: cgcagaaactggaactcctc	26	91	Human
Pml	F: tgtcgacaacaggactctgc<br>R: cctcctgtatggcttgctct	6	112	Mouse
Nfatc1	F: tccaaagtcattttcgtgga<br>R: tttgcttccatctcccagac	50	62	Mouse
Dcstamp	F: tgggggacttatgtgtttcc<br>R: agactcccaaatgctggatg	20	64	Mouse
Ctsk	F: cgaaaagagcctagcgaaca<br>R: tgggtagcagcagaaacttg	18	67	Mouse
Alpl	F: cggatcctgaccaaaaacc<br>R: tcatgatgtccgtggtcaat	31	74	Mouse
Col1a1	F: ctcctggcaagaatggagat<br>R: aatccacgagcaccctga	79	91	Mouse

Table S2. Number of mice (WT and *Pml*$^{-/-}$) scanned by MicroCT and screened for presence of focal PDB-like bone lesions.

**Mouse Model**	**PML knockout**			
**Age group**	**4 months**		**14 months**	
**Genotype**	**WT**	***Pml*$^{-/-}$**	**WT**	***Pml*$^{-/-}$**
**Total (n)**	**8**	**8**	**8**	**8**
**Total with lesion (n)**	**0**	**0**	**0**	**0**
